# Triple-Targeted Therapy With Encorafenib, Binimetinib, and Osimertinib in *EGFR-*Mutated NSCLC With Acquired *BRAF* Alteration After Osimertinib: A Single-Center Experience

**DOI:** 10.1016/j.jtocrr.2026.101056

**Published:** 2026-07-24

**Authors:** Kevin Yijun Fan, Michelle Ku, Beatriz Jimenez Munarriz, Tracy Stockley, Andrew Rabinovitch, Geoffrey Liu, Penelope Bradbury, Natasha B. Leighl

**Affiliations:** a Division of Medical Oncology, University of Toronto, Toronto, Ontario, Canada; b Princess Margaret Cancer Centre, University Health Network, Toronto, Ontario, Canada; c Department of Laboratory Medicine & Pathobiology, University of Toronto, Toronto, Ontario, Canada; d Mackenzie Health Richmond Hill Hospital, Toronto, Ontario, Canada

**Keywords:** Non–small cell lung cancer, BRAF, EGFR, Encorafenib, Binimetinib, Osimertinib

## Abstract

**Introduction:**

Acquired *BRAF* alterations occur in 3% of patients with *EGFR*-mutant metastatic NSCLC who progress on osimertinib and represent a challenging resistance mechanism. Although case series have reported activity with dabrafenib, trametinib, and osimertinib in metastatic NSCLC, there are no published data on the combination of encorafenib, binimetinib, and osimertinib (E+B+O) in this population.

**Methods:**

Between September 2022 and December 2025, eligible patients at the Princess Margaret Cancer Centre with metastatic NSCLC harboring *EGFR* exon 19 deletion or L858R mutations who acquired an actionable or potentially actionable *BRAF* alteration after progression on osimertinib were treated with concurrent E+B+O. Time on therapy, progression-free survival, and overall survival were assessed from the initiation of E+B+O. Response was assessed using Response Evaluation Criteria in Solid Tumors v.1.1. Adverse events were graded using Common Terminology Criteria for Adverse Events v.6.0.

**Results:**

Five patients were included. The median age was 60 (range 56–67) years. All patients had *EGFR* exon 19 deletion at diagnosis. Three patients had acquired *BRAF* V600E, one *BRAF* V600E and D594N, and one *BRAF* G469A variants. One of the five patients achieved a partial response (overall response rate [ORR] 20%) with a duration of response of 32 months and sustained intracranial control. The remaining four patients experienced disease progression. The median time on therapy was 2.0 (range 1.0–39) months, progression-free survival was 2.8 (range 1.7–34) months, and overall survival was 6.6 (range 2.9–39) months. Two patients required dose interruption or discontinuation due to gastrointestinal toxicity (grade 2 diarrhea and grade 3 colitis). No pyrexia was observed.

**Conclusions:**

This study presents a novel treatment combination for *EGFR*-mutant metastatic NSCLC with an acquired *BRAF* alteration. Larger studies are needed to determine whether E+B+O may be considered in carefully selected patients with close response assessment and monitoring for gastrointestinal toxicity.

## Introduction

In patients with metastatic *EGFR*-mutated NSCLC treated with osimertinib, a third-generation tyrosine kinase inhibitor (TKI), progression occurs after a median of 19 months.^1^ Progression is driven by acquired molecular resistance mechanisms, including mutations in v-Raf murine sarcoma viral oncogene homolog B (*BRAF*); most often, *BRAF* V600E occurs in 3% of cases.^2^

*BRAF* V600E is readily targetable in NSCLC using BRAF and mitogen-activated protein kinase (MEK) inhibitors, such as encorafenib and binimetinib and dabrafenib and trametinib. The phase II PHAROS (An Open-label Study of Encorafenib + Binimetinib in Patients With BRAFV600-mutant Non-small Cell Lung Cancer) study of encorafenib and binimetinib in patients with *BRAF* V600E-mutated metastatic NSCLC^3^ demonstrated an objective response rate (ORR) of 75%, progression-free survival (PFS) of 30.2 months, and overall survival (OS) of 47.6 months in treatment-naive patients, with a 22% incidence of pyrexia. In comparison, an earlier phase II study of dabrafenib and trametinib^4^ in a similar population reported an ORR of 64%, PFS of 10.8 months, and OS of 17.3 months in treatment-naive patients, with a 56% incidence of pyrexia.

In patients with *EGFR* and *BRAF* co-mutant NSCLC, there is a biological rationale for pursuing combination therapy targeting both mutations due to pathway crosstalk^5^ and tumor heterogeneity.^6^ Although case series^7^, ^8^, ^9^ have reported the safety and efficacy of combining dabrafenib, trametinib, and osimertinib in patients with *EGFR* and *BRAF* co-mutated metastatic NSCLC, we aim to investigate the efficacy and tolerability of combining encorafenib, binimetinib, and osimertinib in this population, which has not been reported to date. We present this study in accordance with the Strengthening the Reporting of Observational Studies in Epidemiology (STROBE) checklist (https://www.strobe-statement.org/checklists/).

## Methods

Between September 2022 and December 2025, eligible patients at the Princess Margaret Cancer Centre (i.e., adults ≥18 y of age with biopsy-confirmed metastatic NSCLC with *EGFR* L858R or *EGFR* exon 19 deletion) were included if they acquired an actionable or potentially actionable *BRAF* variant on next-generation sequencing (NGS) after progression on osimertinib treatment and were subsequently offered off-label treatment with concurrent encorafenib, binimetinib, and osimertinib. NGS included tissue-based NGS (platforms included Oncomine Comprehensive Assay [Thermo Fisher Scientific] and Oncomine Precision Assay [Thermo Fisher Scientific]) and/or liquid-based NGS (platforms included FoundationOne Liquid Companion Diagnostic [Foundation Medicine], Guardant 360 [Guardant Health], Canexia Follow It [Canexia Health], and, in cases assessing for *EGFR* resistance mutations, targeted ddPCR) (Table 1).

**Table 1:** Baseline Clinical Characteristics and Molecular Alterations

Patient ID	Age, y^a^	Sex	Smoker	Stage at Diagnosis	Site(s) of metastas(es)^b^	PD-L1	Initial Alteration(s)	Alteration(s) on Subsequent Biops(ies)^c^	Type of Biopsy	Previous line(s)^d^	ECOG^e^
1	56	M	N	IV	Lung, bones, brain, choroid, lymph nodes	1%–49%	*EGFR* exon 19 deletion	*BRAF* V600E, 22% **^OC^** *EGFR* exon 19 deletion, 42% **^OC^** *EGFR* T790M^g^, **^D^**	Tissue and liquid	2	1
2	67	M	N	IIIA	Lung, adrenal, bone	<1%	*EGFR* exon 19 deletion, 48% **^OC^**	*BRAF* V600E, 0.2%**^F^** *BRAF* D594N, 0.4%**^F^** *EGFR* exon 19 deletion, 14%**^F^** *KRAS*^f^, **^F^** *FGFR3*– *TACC3* fusion **^F^** *RAF1* E478K, 0.1% **^F^** *TP53* V157D, 0.1% **^F^** *PIK3CA* N345K, 1% **^F^**	Tissue and liquid	1	2
3	62	F	N	IV	Bones	1%–49%	*EGFR* exon 19 deletion	*BRAF* V600E, 0.7% **^G^** *EGFR* exon 19 deletion, 6% **^G^** *EGFR* C797S, 0.2% **^G^**	Tissue and liquid	1	1
4	56	M	4 p.y.	IV	Bones, liver, brain, globes	<1%	*EGFR* exon 19 deletion TP53 K132N, 35% **^OC^**	*BRAF* G469A, 2% **^C^** *EGFR* exon 19 deletion 62% **^C^** *CTNNB1* S37F, 15% **^C^**	Tissue and liquid	1	1
5	60	F	N	IV	Pleura	<1%	*EGFR* exon 19 deletion, 38% **^OP^**	*BRAF* V600E, 13% **^OP^** *EGFR* exon 19 deletion, 51% **^OP^**	Tissue	1	2

C, Canexia Follow It; D, targeted ddPCR; F, FoundationOne Liquid Companion Diagnostic; G, Guardant 360; OC, Oncomine Comprehensive Assay; OP, Oncomine Precision Assay; PD-L1, programmed death-ligand 1; p.y., pack year.

a Age at diagnosis of metastatic disease.

b Site(s) of metastases and Eastern Cooperative Oncology Group at time of initiation of triple therapy.

c Only tier I and II variants are listed. Numbers after molecular alterations indicate variant allele frequencies (VAF), when available.

d Line of therapy for metastatic disease.

e Site(s) of metastases and Eastern Cooperative Oncology Group at time of initiation of triple therapy.

f Various alterations in *KRAS* by FoundationOne Liquid Companion Diagnostic liquid biopsy: G12C (VAF 0.1%), G12D (VAF 5%), G12V (VAF 0.2%), G12A (VAF 4%), G12R (VAF 0.6%), Q61K (VAF 2%), and A59T (VAF 0.6%).

g *EGFR* T790M was detected by targeted ddPCR after progression on afatinib, but was no longer detectable on subsequent biopsy.

Tumor assessments by imaging were conducted at approximately 3-month intervals in accordance with routine clinical practice; however, deviations from the scheduled interval were allowed to accommodate real-world circumstances, including scanner availability and patient clinical condition. Investigator-assessed Response Evaluation Criteria in Solid Tumors (RECIST) v.1.1^10^ evaluations were performed retrospectively through review of serial imaging studies, and when formal measurements were unavailable, radiology interpretation was substituted. Clinical outcomes, including time on therapy (TOT), PFS, and OS, were assessed from the start date of triple-targeted therapy. Adverse events were assessed based on review of clinical documentation and investigations according to Common Terminology Criteria for Adverse Events v.6.0.^11^ Data visualization was performed using RStudio v.2024.12.1 (RStudio, an integrated development environment for R [Posit Software, PBC, 2024]).

## Results

Five patients were treated with concurrent encorafenib, binimetinib, and osimertinib. Their baseline characteristics and molecular alterations are presented in Table 1. The median age at diagnosis of metastatic lung cancer was 60 (range: 56–67) years. Four patients were diagnosed with stage IV disease on initial presentation, and one patient was initially treated with chemoradiation for stage IIIA disease before developing metastatic progression. All patients had EGFR exon 19 deletion on initial biopsy. Three patients had an acquired *BRAF* V600E mutation, one patient had *BRAF* V600E and *BRAF* D594N mutations, and one patient had a *BRAF* G469A mutation. Before triple therapy, four patients received osimertinib monotherapy as first-line palliative therapy and one patient also received afatinib before osimertinib. At the time of initiation of triple therapy, three patients had more than one site of metastatic disease and two patients had central nervous system (CNS) disease (Table 1). Three patients had RECIST v.1.1 measurable disease.

**Figure 1:**
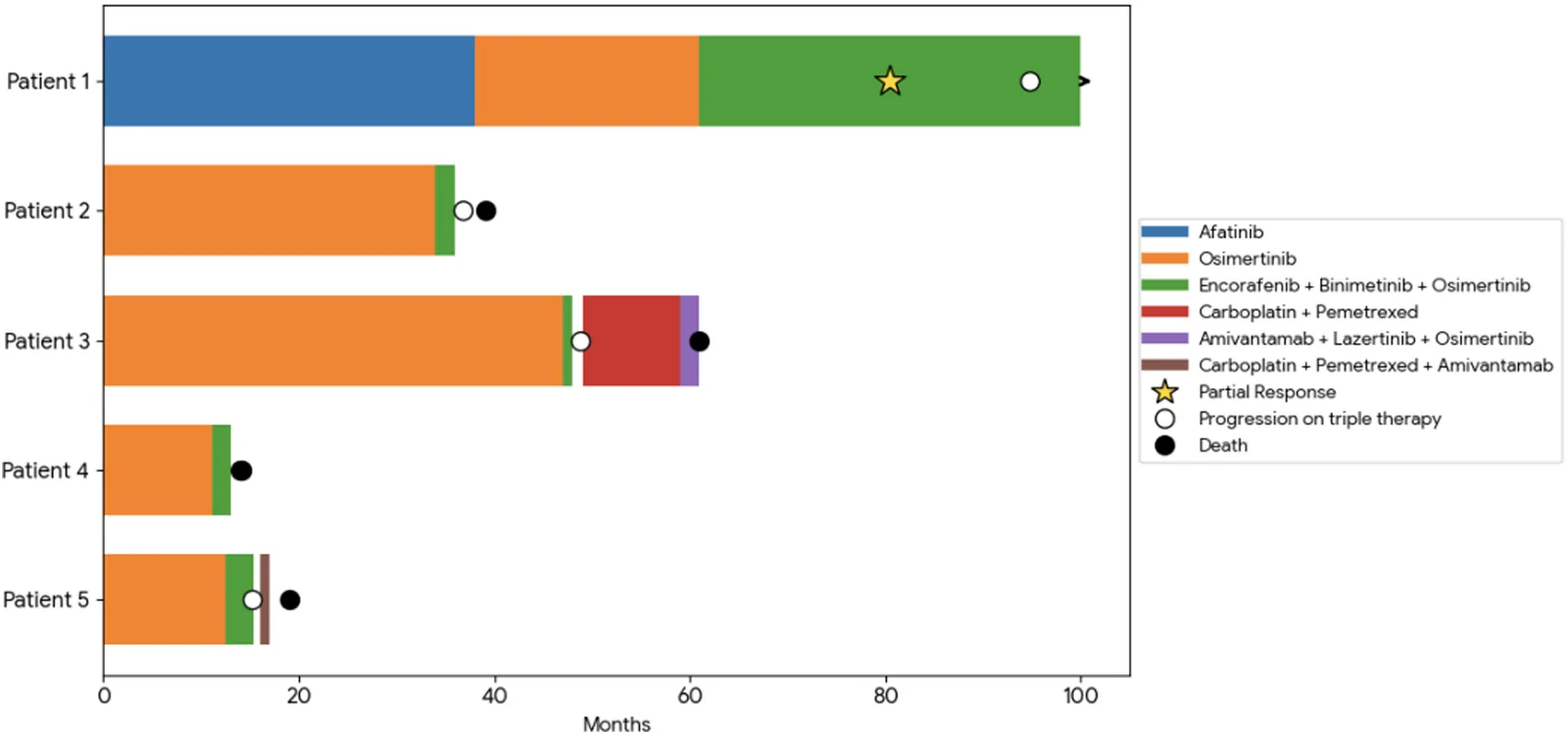
Swimmer plot depicting courses of palliative treatments and clinical outcomes. Time intervals are calculated from the date of initiation of the first systemic therapy for metastatic disease. The colors of the bars represent different types of systemic therapy, as indicated in the legend.

Median follow-up was 6.6 (range 2.9–39) months. Four patients were treated with preemptive upfront dose-reduced encorafenib and binimetinib due to preexisting performance status and co-morbidities (median doses were 300 mg once daily and 30 mg twice daily, respectively), whereas all patients remained on a full dose of osimertinib 80 mg once daily (Table 2). One patient experienced a partial response to triple therapy (i.e., an ORR of 20%), with a duration of response of 32 months and stable intracranial disease (Fig. 1). This patient also had the highest *BRAF* V600E variant allele frequency (VAF) of 22% (Table 1). Four patients experienced disease progression, and two died due to CNS progression. One patient died due to sepsis after receiving subsequent-line chemotherapy, and another patient died due to extracranial progression and pleural effusion; there were no treatment-related deaths. Median TOT was 2.0 (range 1.0–39) months, median PFS was 2.8 (range 1.7–34) months, and median OS was 6.6 (range 2.9–39) months (Table 2 and Fig. 1). The frequency of any-grade adverse events was 80% (four of five patients), and the frequency of grade 3 or higher adverse events was 20% (one of five patients). The most common adverse events were fatigue (3/5 patients) and diarrhea (2/5 patients). Treatment was interrupted in one patient due to diarrhea, which was intermittent grade 1 since starting therapy and was managed with loperamide and diphenoxylate/atropine and became up to grade 2 after 2 years. Treatment was discontinued in another patient due to endoscopically proven grade 3 colitis that occurred 1 month after initiation of therapy, which clinically resolved on discontinuation of treatment. No patients experienced treatment-related pyrexia (Table 2).

**Table 2:** Response to Triple-Targeted Therapy and Treatment-Related Toxicities

Patient ID	Osimertinib Dose	Encorafenib Dose	Binimetinib Dose	Best Response	DoR	CNS Response	TOT	PFS	Site(s) of Progression	OS	Cause of Death	Fatigue	Diarrhea	Colitis	Nausea	Anemia
1	80 mg OD	150 mg OD	30 mg BID	PR	32	SD	39	34	Lung	39	NA (alive)	G1	G2^a^			
2	80 mg OD	225 mg OD	15 mg BID	PD	NA	PD	2.0	2.8	Brain, lung, adrenal, seminal vesicles, lymph nodes	5.2	CNS progression	G1	G1		G1	
3	80 mg OD	300 mg OD	30 mg BID	PD	NA	NA	1.0	1.7	Lung	14	Progression of lung cancer and related pleural effusions			G3^a^		
4	80 mg OD	300 mg OD	30 mg BID	PD	NA	PD	1.9	2.9	Brain	2.9	CNS progression	G1				G2
5	80 mg OD	450 mg OD	45 mg BID	PD	NA	NA	2.9	2.8	Pleura, liver, adrenal, bones	6.6	Sepsis					

*Note*: All time intervals are in months. Response assessments are based on RECIST v.1.1. Grading of adverse events is based on CTCAE v.6.0.

BID, twice a day; CNS, central nervous system; CTCAE, Common Terminology Criteria for Adverse Events; DoR, duration of response; OD, once a day; OS, overall survival; PD, progressive disease; PFS, progression-free survival; PR, partial response; RECIST, Response Evaluation Criteria in Solid Tumors; SD, stable disease; TOT, time on therapy.

a Patient 1 underwent brief treatment interruptions due to grade 2 diarrhea; treatment was discontinued in patient 3 due to grade 3 colitis.

## Discussion

This is the first report of a novel triple-targeted therapy combination with encorafenib, binimetinib, and osimertinib in metastatic NSCLC. Although the ORR was modest, there is a possibility of achieving a durable response. Hence, with more investigation into predictive biomarkers (e.g., *BRAF* mutation VAF), additional studies are needed to determine whether this is a suitable therapeutic consideration in carefully selected patients with close interval monitoring. Several previous studies put these results into perspective. First, the PHAROS clinical trial reported an ORR of 49% and PFS of 9.3 months with encorafenib and binimetinib in previously treated patients with *BRAF* V600E-mutant NSCLC. However, in this trial, no patients had previously received EGFR-directed therapy, and only 10% of patients had baseline brain metastases. Third, the largest case series of dabrafenib, trametinib, and osimertinib combination therapy in *EGFR* and *BRAF* co-mutant NSCLC reported an ORR of 60% and median PFS of 7 months.^8^ However, nine of 12 patients in this case series were identified from a literature review of previously published individual case studies, which are inherently subject to positive publication bias. Finally, a recent literature review demonstrated that in patients with *BRAF* alteration after EGFR TKI, triple-targeted therapy with dabrafenib, trametinib, and osimertinib was associated with a significantly longer PFS compared with other treatments (8.0 versus 2.5 mo); however, only half of the patients in this study were resistant to a third-generation EGFR TKI.^12^

With respect to safety and tolerability, treatment interruption or discontinuation occurred in two patients, both due to gastrointestinal toxicity, which included intermittent grade 2 diarrhea and early-onset grade 3 colitis. Given that diarrhea has been reported in 44% of patients treated with encorafenib and binimetinib^3^ and in 58% of patients treated with osimertinib,^13^ it is possible that gastrointestinal toxicity may be additive when these agents are administered in combination. This highlights the need for further studies to determine the optimal dosing strategy for this triple therapy combination.

Limitations include the small sample size and single-center design. We did include one patient with a class II *BRAF* mutation (i.e., *BRAF* G469A). Although phase I/II clinical data have demonstrated activity of encorafenib and binimetinib in patients with solid tumors harboring this alteration,^14^ the heterogeneity of our cohort (i.e., inclusion of non-V600E alterations in *BRAF* and additional co-alterations) is a limitation of our study design. In addition, patients underwent different types of molecular testing (e.g., blood versus tissue-based; different genomic panels) due to variability in the time and setting of testing and feasibility of obtaining biopsy material in some cases. In patients in whom more than one clinically targetable mutation is found, future studies are required to determine the sequence of targeting these mutations. Because all patients with acquired *BRAF* alteration after osimertinib during the study time period were offered treatment with triple-targeted therapy, due to the rarity of this molecular profile, it was not possible to conduct a matching paired comparison with patients treated with cytotoxic therapy. In addition, given that first-line therapy for *EGFR-*mutant NSCLC now includes intensification strategies,^15^^,^^16^ resistance mechanisms after these treatments may differ, and future studies will need to address this. In the future, there is a need for interinstitutional collaboration to investigate larger cohorts of patients with this rare molecular profile. For example, the ORCHARD (Osimertinib plus datopotamab deruxtecan in patients with EGFR-mutated advanced NSCLC after progression on first-line osimertinib) trial (NCT03944772) includes a cohort of patients with NSCLC with *EGFR* and *BRAF* alterations treated with osimertinib and selumetinib. Our findings warrant validation in larger prospective studies.

## CRediT Authorship Contribution Statement

**Kevin Yijun Fan:** Conceptualization, Data curation, Formal analysis, Investigation, Methodology, Software, Validation, Visualization, Writing - original draft, Writing - review & editing.

**Michelle Ku:** Data curation, Resources, Writing - review & editing.

**Beatriz Jimenez Munarriz:** Writing - review & editing.

**Tracy Stockley:** Writing - review & editing.

**Andrew Rabinovitch:** Investigation, Resources, Writing - review & editing.

**Geoffrey Liu:** Investigation, Resources, Writing - review & editing.

**Penelope Bradbury:** Investigation, Resources, Writing - review & editing.

**Natasha B. Leighl:** Conceptualization, Funding acquisition, Investigation, Methodology, Project administration, Resources, Supervision, Validation, Visualization, Writing - original draft, Writing - review & editing.

## Disclosure

The authors declare no conflict of interest.
