# Supplementary material for: Triple-Targeted Therapy With Encorafenib, Binimetinib, and Osimertinib in EGFR-Mutated NSCLC With Acquired BRAF Alteration After Osimertinib: A Single-Center Experience
Source: JTO Clin Res Rep. 2026 Jul 24;7(10):101056. doi: 10.1016/j.jtocrr.2026.101056 (PMC13579456; doi:10.1016/j.jtocrr.2026.101056)
Supplement: Supplementary file 1 [file mmc1.docx]

**Supplement:**

**Individual patient summaries:**

**Patient 1:** A 56-year-old man, never smoker, presented with bone pain and was diagnosed with lung adenocarcinoma, PD-L1 1-49%, with *EGFR* exon 19 deletion, metastatic to the brain, bones and bilateral choroids. He received palliative radiation to the brain and bones, then started on first line afatinib 40 mg once daily, with partial response. He was also started on denosumab. He had sustained intracranial complete response as well as no further evidence of disease in the eyes on serial ophthalmological assessments, despite not having received local therapy to the eyes. Subsequent biopsy on progression in the lungs demonstrated *EGFR* T790M, and his treatment was switched to osimertinib 80 mg once daily. ctDNA approximately one year later demonstrated demonstrated persistence of *EGFR* exon 19 deletion, with *EGFR* T790M no longer detectable. Upon progression in lungs and thoracic lymph nodes, fine needle aspiration of a subcarinal lymph node demonstrated *BRAF* V600E mutation, with persistent *EGFR* exon 19 deletion. He then started encorafenib 150 mg once daily and binimetinib 30 mg BID, while continuing on osimertinib 80 mg daily. Of note, this was a pre-emptive upfront dose reduction due to a recent unrelated admission for hypotension and supraventricular tachycardia and was not subsequently escalated due to diarrhea; he initially experienced intermittent grade 1 diarrhea since starting treatment, which was managed with loperamide and diphenoxylate/atropine, then experienced persistent grade 2 diarrhea two years later which resulted in treatment interruption. CT (computer tomography) imaging two months later demonstrated significant improvement of diffuse parenchymal and pleural modularity as well as decrease in size of mediastinal lymph nodes, and there was a new small sclerotic bone lesion with otherwise unchanged bone metastases, overall representing partial response (Figure S1). He also experienced grade 1 fatigue, however he remained Eastern Cooperative Oncology Group (ECOG) performance status 1 throughout treatment. Two years and ten months into triple therapy, he developed progression in lung lesions, however the decision was made to continue treatment due to the small volume of disease and stable areas of disease elsewhere. He currently remains on treatment.

**Supplemental Figure S1. CT chest prior to (left panel) and after two months of treatment with triple targeted therapy (right panel) in Patient 1, demonstrating partial response.**

**Patient 2:** A 66-year-old man, never smoker, presented with persistent cough, was initially diagnosed with stage IIIA (T1a N2) lung adenocarcinoma, PD-L1 <1%. Initial biopsy demonstrated *EGFR* exon 19 deletion. He underwent chemoradiation of the mediastinum, 60 gray in 30 fractions with concurrent carboplatin and paclitaxel. He proceeded with three cycles of consolidation durvalumab which was stopped due to pneumonitis. Four months later, he started osimertinib 80 mg daily due to low volume metastatic disease in the adrenal gland and ilium. He also started zoledronic acid at this time. He experienced a partial response, and eventually experienced progression in the lung after nearly three years of targeted therapy, for which he underwent urgent radiotherapy due to hemoptysis. Liquid biopsy at this time demonstrated *BRAF* V600E mutation, persistent *EGFR* exon 19 deletion, and several other alterations on blood-based next generation sequencing (Table 1). Later that month, he initiated encorafenib 225 mg daily and binimetinib 15 mg BID, while continuing osimertinib 80 mg; pre-emptive dose reduction was due to frailty. He experienced grade 1 fatigue, grade 1 nausea, grade 1 diarrhea, and remained ECOG 2 throughout treatment. This treatment was discontinued three months later due to widespread progression in the lung, adrenal, seminal vesicles, cerebellum, and lymphadenopathy in the abdomen, pelvis and supraclavicular areas. He underwent urgent craniotomy for resection of a large posterior fossa mass. Shortly afterwards, he was admitted to the palliative care unit and passed away, four years and three months after initial diagnosis.

**Patient 3:** A 62-year-old woman, never smoker, presented with persistent cough, and was diagnosed with stage IV lung adenocarcinoma, PD-L1 1-49%, harbouring *EGFR* exon 19 deletion, metastatic to the bones. She started osimertinib 80 mg daily with partial response. Zoledronic acid was started the following year. Over three years later, she developed progression in the bones, and underwent liquid biopsy which identified *BRAF* V600E mutation, *EGFR* C797S mutation, and persistent *EGFR* exon 19 deletion. She received several courses of palliative radiotherapy to the pelvis, thoracic and lumbar spine. She then she started encorafenib 300 mg daily and binimetinib 30 mg BID, in addition to continuing osimertinib 80 mg. One month later, she was admitted for hematochezia, with colonoscopy identifying hyperpigmentation, friable mucosa and petechiae. Her biopsies revealed active inflammatory changes in the colon and ileum, these findings were felt to be highly suggestive of treatment-related colitis and ileitis. At this time, she also underwent restaging which demonstrated progression with new lung nodules, with stable bony disease. She then completed four cycles of carboplatin and pemetrexed, followed by four cycles of maintenance pemetrexed. She continued on osimertinib during this time. Repeat liquid biopsy redemonstrated *BRAF* V600E mutation, *EGFR* C797S mutation, and *EGFR* exon 19 deletion. She then started amivantamab with lazertinib. She was admitted to hospital the following month, initially for unstable upper gastrointestinal bleeding, then located pleural effusion, with imaging showing ongoing disease progression. This was already twelve months after her aforementioned episode of colitis. Esophagogastroduodenoscopy demonstrated bleeding angioectasias, severe EGD showed bleeding angioectasias, severe esophagitis, and gastric ulcers, treated with clips and argon plasma coagulation. Her upper gastrointestinal bleeding then resolved, and she did not have further gastrointestinal bleeding for the following two weeks of her admission up until her death. During this time she developed other medical issues, including loculated pulmonary infection with pleural effusion, and repeat CT imaging showed progression of her lung cancer. Eventually, she was transitioned to best supportive care and passed away in hospital, at five years after diagnosis. The cause of death was documented to be progressing lung cancer and related pleural effusions.

**Patient 4:** A 56-year-old man with four pack-year remote smoking history presented with vision changes, and was diagnosed with lung adenocarcinoma, *EGFR* Exon 19 deletion, PD-L1 <1% of the left upper lobe, widely metastatic to the brain, bilateral globes, and bones. He received radiotherapy to bilateral globes, shoulder, pelvis, and tibia, followed by whole brain radiotherapy. He was then started on osimertinib 80 mg daily and experienced a partial response. He received re-irradiation to the right globe five months later due to oligoprogression. Nine months after starting osimertinib, he developed progression in the bones, with a soft tissue mass near acetabulum, and the liver; biopsy of the pelvic soft tissue mass demonstrated *BRAF* G469A, and redemonstrated *EGFR* exon 19 deletion. He underwent re-irradiation of the spine and pelvis. He then began triple therapy with encorafenib 300mg daily and binimetinib 30mg BID, while continuing osimertinib 80 mg daily; pre-emptive dose reduction was due to anemia. He also received zoledronic acid. He developed grade 1 fatigue and grade 2 anemia, and remained ECOG 1. Two months into this treatment, he developed neurological symptoms (i.e. fall, confusion) while travelling out of country, and discontinued encorafenib and binimetinib while continuing osimertinib. Upon return to Canada, MRI (magnetic resonance imaging) of the brain demonstrated progression of brain metastases with leptomeningeal involvement. He underwent repeat whole brain radiotherapy, and passed away shortly afterwards, at one year and four months after diagnosis.

**Patient 5:** A 60-year-old woman, never smoker, initially presented with abdominal discomfort and dyspnea, and was diagnosed with metastatic lung adenocarcinoma, PD-L1 <1%, with *EGFR* exon 19 deletion based on targeted molecular testing of malignant pleural effusion. She did not have other sites of distant metastatic disease. She was treated with osimertinib 80 mg daily, with partial response. Eleven months later, she developed disease progression in the pleura requiring catheter insertion, with pleural fluid demonstrating *BRAF* V600E mutation along with persistent *EGFR* exon 19 deletion. She then initiated encorafenib 450 mg and binimetinib 45 mg BID, while continuing osimertinib 80 mg daily. She remained ECOG 2 throughout treatment, and tolerated treatment well without toxicities. Three months later, she developed disease progression in the pleura, liver, adrenal, and bones. She was switched to carboplatin, pemetrexed and amivantamab. After one month, she had ongoing disease progression in the lungs, liver, bones, and lymph nodes causing hydronephrosis. She then had several hospital admissions for sepsis and hypotension, before passing away one year and seven months after diagnosis.
